# Understanding the Transcriptional Changes During Infection of *Meloidogyne incognita* Eggs by the Egg-Parasitic Fungus *Purpureocillium lilacinum*

**DOI:** 10.3389/fmicb.2021.617710

**Published:** 2021-04-07

**Authors:** Wen-Feng Xu, Jia-Lin Yang, Xiang-Kun Meng, Zhi-Guang Gu, Qi-Lin Zhang, Lian-Bing Lin

**Affiliations:** ^1^Faculty of Life Science and Technology, Kunming University of Science and Technology, Kunming, China; ^2^Kingenta Ecological Engineering Group Co., Ltd., Linyi, China; ^3^Engineering Research Center for Replacement Technology of Feed Antibiotics of Yunnan College, Kunming, China

**Keywords:** Nematophagous fungus, RNA-seq, transcriptomics, *Meloidogyne incognita* eggs, gene expression, *Purpureocillium lilacinum*

## Abstract

The egg-pathogenic fungus *Purpureocillium lilacinum* parasitizes on nematode eggs, and thus, it is used as a good biocontrol agent against plant root-knot nematodes. However, little is known about the transcriptional response of *P. lilacinum* while infecting nematode eggs. This study presents the whole transcriptome sequencing of *P. lilacinum* and transcriptome-wide gene expression analysis of *P. lilacinum* upon infecting the eggs of *Meloidogyne incognita* compared to non-infecting controls. A transcriptomic library of *P. lilacinum* was used as reference gene set and six transcriptomic libraries of the non-infecting control and *P. lilacinum* infecting *M. incognita* eggs were constructed, respectively, comprising three biological replicates of each. A total of 1,011 differently expressed genes (DEGs) were identified in the infecting samples, including 553 up-regulated and 458 down-regulated genes compared to the non-infecting control samples. Furthermore, functional enrichment analysis exhibited that these DEGs were primarily involved in oxidative phosphorylation, oxidoreductase activity, and metabolic processes. Fifteen DEGs were randomly selected to verify the RNA sequencing results through quantitative real-time polymerase chain reaction (qPCR). The study focused on *P. lilacinum* genes that were strongly expressed upon infecting *M. incognita* eggs. These DEGs were primarily involved in detoxification, parasitic behavior, and nutritional utilization. This study contributes significantly to the understanding of the molecular mechanisms underlying the parasitic action of *P. lilacinum* on nematode eggs and provides a valuable genetic resource for further research on parasitic behavior of *P. lilacinum*. Notably, this study examined the transcriptomics of *P. lilacinum* infecting *M. incognita* eggs at only one time point. Since there were fungi at different stages of the infection process at that time point, the transcriptional profiles are not precisely examining one specific stage in this process.

## Introduction

Plant parasitic nematodes (PPN) cause major damage to a variety of crop species worldwide (Moens and Perry, 2009). In particular, root-knot nematodes have been most widely investigated because of their economic importance and their dominant position among PPN (Jones et al., 2013; Kyndt et al., 2016; Chen et al., 2017). *Meloidogyne* is globally one of the most important genera of root-knot nematodes. Plant damage caused by *Meloidogyne* is most severe in temperate, subtropical, and tropical regions, and diseases induced by *Meloidogyne* infestation result in average decrease in crop yield by 10–20%, which increases up to 75% in severely infected cases (Liang and Zheng, 2010). In response to increasing prohibition of chemical pesticides, development of environmentally friendly alternative control methods for PPN is urgently required. As a safe and effective approach, biological methods have been widely studied and applied for controlling PPN (Na et al., 2015; Poveda et al., 2020). For the biological control of PPN using fungi, most endoparasitic and opportunistic fungal species have already been developed into mature products (Na et al., 2015). Endoparasitic and opportunistic fungi can capture nematodes by either absorption or adhesion to the body surface or to the eggs of nematodes by recognizing the host. Under the action of chitinase, the fungal mycelium penetrates the body wall and digests the eggshell, thus further, the eggs and larvae of PPN are infected and the fungal mycelium further propagates (Na et al., 2015). Such fungi primarily include the genera *Purpureocillium*, *Pochonia*, and *Hirsutella*.

*Purpureocillium lilacinum* is a popular nematode egg parasite and can infect both larvae and adults, which are abundant in the soil, in particular, in the rhizosphere of plants (Na et al., 2015). Many prominent examples exist in literature reports where *P. lilacinum* was employed as a biological control for nematode eggs. For example, spore suspension of two strains of *P. lilacinum* (PLA and PLB) isolated from roots and rhizosphere soils of black pepper exhibited 66.0 and 78.8% parasitism on eggs respectively. Hatching of *Meloidogyne incognita* eggs incubated in spore suspension of PLA and PLB was significantly reduced for seven days, with inhibition of hatching of 88–89% of eggs compared to only 26% in the non-incubated control (Guan Pau Chen et al., 2012). Currently, *P. lilacinum* is widely used in commercial preparations for the biological control of PPN as well as for controlling other pathogenic nematodes that severely infect economically important crops (Li et al., 2015; Na et al., 2015).

Despite their significant use for the biological control of PPN, only few studies have explored the molecular mechanisms underlying the infection of PPN by *P. lilacinum*. Moreover, the existing related studies have extensively focused upon the morphology and enzymology. For instance, a serine protease and a prepared enzyme (consisting of six chitinases from a liquid culture of *P. lilacinum*) were applied to the eggs of the nematode *Meloidogyne javanica* to assess the effect of these enzymes on eggshell structures (Khan et al., 2004). The results indicated that the use of a combination of *P. lilacinum* proteases and chitinases caused major changes in the eggshell structures of *M. javanica*. These changes included the destruction of the lipid and the chitin layers, as well as the loss of the integrity of the vitelline layer. Moreover, *P. lilacinum* protease and chitinase enzymes, either individually or in combination, caused a reduction in the hatching rates of *M. javanica* juveniles (Khan et al., 2004). A basic serine protease from *P. lilacinum*, exhibiting biological activity against eggs of the nematode *Meloidogyne hapla*, showed that serine proteases contributed to the penetration of the mycelium through the egg-shell of nematodes (Peter et al., 1995).

At genomic/gene levels, a number of genes in *P. lilacinum* has been found to be associated with virulence and fungal development (Wang et al., 2010; Yang et al., 2011). Nonetheless, the molecular mechanisms underlying parasitism and the development of genetic resources of *P. lilacinum* have rarely been investigated till date. For example, the genome of a *P. lilacinum* strain isolated from tannery sludge in India was sequenced and analyzed (Prasad et al., 2015). Their study reported the fundamental characteristics of the *P. lilacinum* genome and they further explored its evolutionary relationship. Moreover, the study by Prasad et al. (2015) found gene sets encoding G-protein coupled receptors, proteases, glycoside hydrolases, and carbohydrate esterases in the *P. lilacinum* genome as well as a variety of secondary metabolites. It has been suggested that these gene sets are extremely important for the degradation of nematode-egg shell components and adaptation to heterogeneous lifestyles. Later, Xie et al. (2016) sequenced the draft genome and the transcriptome of *P. lilacinum* strain 36-1 (isolated from the surface of the eggs of *M. incognita* from soil). Further comparative evaluation of genome and transcriptome analyses aided in identification of the specific parasitism of *P. lilacinum* and identified the genes responsible for the infection of nematode eggs. Notably, these studies focused on the general features of the genome as well as the genome-based phylogeny of *P. lilacinum*, specific gene/protein families, and signal transduction genes. However, the gene functional modules and pathways related to the infection, as well as genes with a strong expression response to the infection have not been discussed, comprehensively.

In this study, the transcriptional response of *P. lilacinum* on infection of *M. incognita* eggs at the whole transcriptome level was explored. Moreover, *P. lilacinum* genes involved in the infection of *M. incognita* eggs were identified and the functional importance of functional modules and pathways enriched by these genes were also identified. RNA-seq was employed to generate transcriptomes of *P. lilacinum*, comparing those with and without infecting *M. incognita* (i.e., treatment vs control). The results were analyzed by using bioinformatics and quantitative real-time polymerase chain reaction (PCR) (qPCR) approaches.

## Materials and Methods

### Sample Collection

Healthy spores of *P. lilacinum* strain JZD02 (isolated from the surface of the eggs of *M. incognita* in the field rhizosphere soil of *Lycopersicum esculentum*, Sichuan Province, China) and *M. incognita* race 1 eggs were provided by Shandong Jinzhengda Ecological Engineering Co., Ltd., Kingenta, China^1^. *M. incognita* was preserved and cultured in the roots of tomato plants at room temperature, and *P. lilacinum* was cultured on potato dextrose agar liquid medium at 25°C. The experimental samples were maintained at the Faculty of Life Science and Technology, Kunming University of Science and Technology in Chenggong, Kunming, Yunnan Province, China. The respective rDNA-ITS molecular markers and BLAST alignment (NCBI) were used to further confirm fungal species according to previous methods (Li et al., 2020). Briefly, 15 individual nematodes were randomly selected and placed in sterile PCR tubes with a volume of 10 μL. All samples were ground using sterilized glass rods in liquid nitrogen. Subsequently, 8 μL 10× PCR buffer (Takara, Japan) and 2 μL protease K (20 mg/mL) were added. The contents were mixed thoroughly, and the mixture was incubated at 65°C for 90 min, followed by incubation at 95°C for 10 min. Further, the samples were centrifuged at 8,000 × *g* for 1 min, and the supernatant was used for PCR amplification. Commonly used primers reported in previous studies (Rengang et al., 2007; Zeng et al., 2010) were employed herein. The PCR reaction system (50 μL) included 2× Taq Mastermixture (25 μL), DNA template (30 ng), and 0.4 mM each of the forward and reverse primers. PCR reactions including pre-denaturation were conducted at 94°C for 5 min, this was followed by 35 cycles of denaturation at 94°C for 30 s, annealing at 58°C for 30 s, extension at 72°C for 1.5 min, and a final extension at 72°C for 10 min. Next, the PCR products were sequenced by Sangong Biotech Co., Ltd. (Shanghai, China). After stitching and manual proofreading, the sequences were submitted to the GenBank database for homolog analysis by using the BLAST online server. Species with the best match and more than 99% homology in the database were confirmed as experimental nematode species.

*Purpureocillium lilacinum* infection of *M. incognita* eggs was initiated by employing the water agar plate method following previously reported protocols with minor amendments (Liu et al., 2005; Ma et al., 2015). Briefly, *M. incognita* egg suspension (500 μL, concentration: 200 eggs/mL) and *P. lilacinum* spore suspension (50 μL, concentration: 2 × 10^8^ spores/mL) were thoroughly mixed and gently coated on the water agar plate, following which the suspension was incubated (Sanyo, Japan) at 28°C for 6 h. The samples were then randomly collected using a hole puncher with 1 cm diameter to estimate the infection rate of *P. lilacinum* on *M. incognita* eggs (counted by 100 eggs) under a DM1000 microscope (Leica, Germany). Noteworthy, a sample was considered to be infected when at least one mycelium had penetrated at least one egg. The magnification was 400 times the original size. These sample collection experiments were repeated every 12 h up to 72 h, with three independent biological replicates each.

**FIGURE 1:**
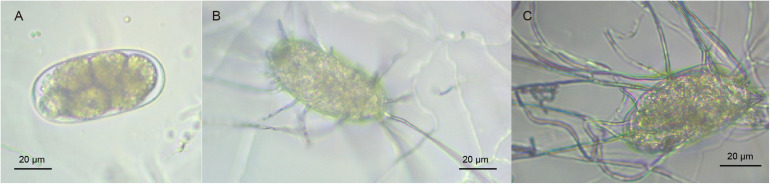
**(A)** Normal *M. incognita* eggs. **(B,C**) *M. incognita* eggs infected due to *P. lilacinum* parasitism.

Supplementary Table 1 presents that the infection rate of *P. lilacinum* gets saturated 42 h post-infection (hpi, ranging from 62 ± 8.1% to 66 ± 6.6%). This indicates that adequate infection occurs at 42 hpi, with maximum *P. lilacinum* mycelium activity compared to other time points. The results indicated that at 6 hpi, the infection rate of *P. lilacinum* was 0%, and no contact was detected between *P. lilacinum* mycelium and *M. incognita* eggs. *P. lilacinum* mycelium containing *M. incognita* eggs was carefully collected at 42 hpi [infected/treated groups (PLT)] by cautiously scraping the surface of plates. For comparative analysis, samples at 6 hpi were processed as non-infected/control groups (PLCK) (Figures 1A–C). The experiment was repeated three times independently, yielding three biological replicates (PLT1, PLT2, and PLT3 as treated groups, as well as PLCK1, PLCK2, and PLCK3 as control groups). Moreover, *P. lilacinum* (PL) only (without *M. incognita* eggs) was used for generating the reference transcriptome for further quantification of gene expression. All samples were frozen immediately in liquid nitrogen and stored at −80°C.

### RNA Extraction and Sequencing

Total RNA was extracted from PL, PLT1–3, and PLCK1–3 samples using RNeasy Mini Kit (Qiagen, Germany) according to manufacturer’s manuals. Residual genomic DNA was removed using an RNase-free DNase kit (Qiagen, Germany). Concentrations of RNA were estimated using a NanoDrop ND1000 spectrophotometer (Thermo Scientific, United States). RNA quality (RNA integrity number and its threshold is seven) was confirmed using a bioanalyzer (Agilent 2100, Agilent Technologies, United States). Library construction from control and treated groups was performed at Novogene Co, Ltd. (Novogene, China) using the Illumina RNA Sample Preparation Kit (Illumina, United States) following the manufacturer’s protocol. Six RNA-seq libraries (containing three biological replicates for treatment (PLT1–3) and three control (PLCK1–3) groups) were constructed and used for comparative analysis of gene expressions. Furthermore, two RNA-seq libraries (PL1 and 2) were constructed and used to build reference gene sets. These eight libraries with paired-ends of a length of 150 bp were sequenced on an Illumina HiSeq 4000 platform (Illumina, United States).

### Quality Control of Data and Transcriptome Assembly of *P. lilacinum*

Raw reads generated based on sequencing were filtered. Adapters, reads with unknown bases (N) > 5%, and low-quality sequences [bases with quality score (Q20) < 20; more than 50% of read sequences] were removed by using *FastQC* software (v 0.11.9) (Brown et al., 2017). To obtain the complete reference gene sets, *Trinity* (Grabherr et al., 2011) was employed to perform *de novo* assembly of mixed clean read pools generated by combining two libraries (PL1–2). Large datasets generated by mixed reads from multiple RNA-seq libraries can improve probability and sensitivity for detection of low-abundance transcripts, as noticed in a recent study (Zhang et al., 2019). Next, the TGICL pipeline (Pertea et al., 2003) was used to discard redundant sequences from the *Trinity* assembly by fast clustering, thus generating the final set of unigenes. To complete the functional annotation of unigenes, local BLASTx was used to search all unigenes in non-redundant (NR), gene ontology (GO), Kyoto Encyclopedia of Genes and Genomes (KEGG), and Swiss-Prot databases. To assess the completeness of the assembly, the unigenes were mapped to a core set (fungi_*odb9*) of fungi by using (Benchmarking Universal Single-Copy Orthologs (BUSCO) v4.1.2 (Seppey et al., 2019). The completeness of the unigene set was automatically evaluated, and the percentages of complete, fragmented, and missing genes were calculated through automatic output of the BUSCO software.

### Identification and Analysis of SSR and SNPs

Simple sequence repeats (SSRs)/microsatellites have been widely used in comparative genomics and fungal genetics (Qiao et al., 2018). SSR distributed on all the obtained PL unigenes were detected by using MIcro SAtellite (MISA) (v1.0) tool (Thiel et al., 2003), and then SSR specific primers were designed by using Primer3 software (Koressaar and Remm, 2007). Moreover, multiple alignments of clean reads corresponding to unigenes were implemented by using HISAT (v0.1.6-beta) (Kim et al., 2015). Next, procedure including the detection and filtering of low-quality single nucleotide polymorphisms (SNPs) was performed by GATK analysis (v3.4-0) (McKenna et al., 2010).

### Analysis of Differentially Expressed Genes

To remove unwanted sequences derived from *M. incognita* eggs, clean reads of PLT1–3 and PLCK1–3 were mapped to the *M. incognita* reference genome downloaded from the NCBI database^2^ by using Bowtie 2 (Langmead et al., 2009) with default parameters (allowing maximum mismatch number, −v 1). Unaligned reads were retained as bona fide *P. lilacinum* sequences for further analysis. To quantify the levels of gene expression, Bowtie 2 (Langmead et al., 2009) was used first to map clean reads of each library from the control and treated groups (i.e., PLT1–3 and PLCK1–3) to the reference unigene set of *P. lilacinum* transcriptome under strict criteria (allowing no mismatch number, −v 0). Subsequently, based on the number of mapped reads on each unigene, the fragments per kilobase of unigenes per million (FPKM) were calculated by using the RNASeq by expectation maximization tool (Conesa et al., 2016). Reproducibility of samples was evaluated among three biological replicates based on the similarity of their unigene expression levels. Furthermore, for studies investigating biological replicates, DESeq software (Anders and Huber, 2010) in the R package is commonly used for the identification of DEGs between two groups, attributed to its low number of false rates (Behringer et al., 2015; Zhang et al., 2019). Therefore, DESeq was used to identify the DEGs between control/PLCK and treated/PLT groups. The threshold for identification of DEG was determined as follows: fold changes (FC) ≥ 2 (|log2 ratio| ≥ 1) and false discovery rate (FDR) values of 0.01 obtained by correcting *p*-values (Wald test in DESeq) by using the Benjamini–Hochberg method (Ferreira and Zwinderman, 2006).

In order to verify the RNA-seq results, qPCR analysis of 15 randomly selected DEGs, including eight up-regulated and seven down-regulated DEGs in treatment groups, was carried out using the same RNA samples that were also used for RNA-seq. The β*-actin* gene was selected as optimal reference gene (Zampieri et al., 2014). Beacon Designer 7 was used to design specific primers for qPCR (Supplementary Table 2). SYBR Prime Script RT-PCR Kit II (Takara, Japan) was used to synthesize the first cDNA chain. Subsequently, RNase-free water was added to the synthetic cDNA until a final concentration of 100 ng/μL was obtained. For qPCR, SYBR Premix Ex Taq II Kit (Takara, Japan) was used, following the manufacturer’s recommendations in an ABI PRISM 7300 fast real-time PCR system (Applied Biosystems, United States). The reaction mixtures with a volume of 10 mL contained 2× SYBR Premix Ex Taq (5 mL), cDNA (1 μL), 0.2 μL each of the 10 mM forward and reverse primers, ROX (0.2 μL), and PCR-grade water (3.4 μL). The qPCR was performed under the conditions including an initial denaturation step at 94°C for 5 min, followed by 40 cycles at 94°C for 1 min, 60°C for 35 s, and 72°C for 40 s. The qPCR reaction was repeated three times for each gene in each sample, and experiments were performed in triplicate (i.e., three biological replicates). Data analysis and statistics were conducted via the 2^–ΔΔ*CT*^ method (Livak and Schmittgen, 2001) by using IBM SPSS Statistics 22 software (IBM Software Inc., Armonk, NY, United States).

The final results are presented as mean ± standard deviation (mean ± SD) of fold changes (PLT vs. PLCK samples) for three independent replicates. Statistical significance of fold changes between PLT and PLCK samples was determined by unpaired two-tailed *t*-test by using IBM SPSS 22.0 software. Differences were considered significant at *p* < 0.01.

### Functional Enrichment Analysis of Differentially Expressed Genes

To further identify the main biological, cellular and molecular functions, as well as the signaling pathways in which the DEGs participated, GO and KEGG annotations of DEGs were extracted by using custom Perl scripts. Subsequently, Blast2GO pipeline (Conesa et al., 2005) was used to perform GO enrichment analysis of DEGs, and Fisher’s Exact test was used in the pipeline to calculate the level of significance for the enrichment of each GO term. Redundant GO terms obtained by enrichment analysis were removed by using the GO trimming software (Jantzen et al., 2011). Furthermore, KOBAS 2.0 (Xie et al., 2011) software was used to enrich KEGG pathways and test the statistical significance for these terms. Noteworthy, the Benjamini–Hochberg adjustment was used to correct the *p*-values of enriched GO and KEGG terms. The GO terms with FDR values <0.05 were retained as final results, and the KEGG terms with FDR values <0.01 were considered as significantly enriched pathways.

## Results

### Overview of RNA-seq Data

A total of 91,440,540 raw reads was obtained for the reference unigene sets of *P. lilacinum* from the mixed PL1 and PL2 data (Table 1). Among these, 92.81% (12.73 Gb) was identified as clean reads after filtering, with an average GC content of 60.84%. This clean data set of the *P. lilacinum* transcriptome used as reference was submitted to the Sequence Read Archive (SRA) of NCBI (Accession No. SRR10017427). The assembled transcriptome was found to contain 23,181 transcripts and 11,858 unigenes, with a mean unigene length of 1,800 bp and an N50 value of 2,714 bp. The results of BUSCO analysis revealed that in the *P. lilacinum* transcriptome assembled herein, 75.3% of the unigenes was “complete,” 12.6% was “fragmented,” and the remaining genes were “missing.” Functional annotation results showed that 9,569, 5,509, 6,850, and 3,385 unigenes were annotated to NR, Swiss-Prot database, GO, and KEGG database, respectively. A total of 10,037 (84.64%) unigenes obtained functional annotation.

**TABLE 1:** Summary of the *Purpureocillium lilacinum* transcriptome generated by using Trinity assembler software.

**Item**	**Number**
Number of raw reads	91,440,540
Number of clean reads	84,869,688
Clean bases (Gb)	12.73
Q20 (%)	98.41
GC content (%)	60.84
Total transcripts	23,181
Total unigene length(nt)	21,348,008
Total unigene number	11,858
Unigene N50 (bp)	2,714
Mean unigene length (nt)	1,800
NR annotation (%)	95,69 (80.69%)
Swiss-Prot annotation	5,509 (46.45%)
GO annotation	6,850 (57.76%)
KEGG annotation	3,385 (28.54%)
All annotated unigenes	10,037 (84.64%)

Based on the control and treated transcriptomes derived from *P. lilacinum* used for the identification of DEGs (Table 2), 275,181,826 raw reads were generated through RNA-seq. After quality control, a total of 265,467,254 (96.47% of total raw data) clean reads for all the six libraries, with sizes ranging from 6.27 to 7.59 Gb, were obtained. These clean reads were submitted to the SRA database of NCBI (Accession numbers: SRR10017428-33). The percentage of total mapped clean reads ranged from 75.64 to 82.67% for the six libraries. The Q20 read percentage of each library accounted for ∼97% of all clean data from each dataset. A high mapping rate and high Q20 percentage of clean reads indicated that the RNA-seq datasets were robust.

**TABLE 2:** Summary of RNA-seq information for each sample from the control and treatment groups. *P. lilacinum* infecting *M. incognita* eggs in three biological replicates (PLT1–3); *P. lilacinum* (controls) in three biological replicates (PLCK1–3).

**Category**	**Controls**			***P. lilacinum* infecting *M. incognita* eggs**		
	**PLCK1**	**PLCK2**	**PLCK3**	**PLT1**	**PLT2**	**PLT2**
Total raw reads	43,313,830	44,094,988	53,109,280	43,497,648	44,398,070	46,768,010
Total clean reads (%)	41,820,460 (96.6)	42,903,842 (97.3)	50,576,966 (95.2)	42,305,064 (97.3)	42,789,346 (96.4)	45,071,576 (96.4)
Total mapped reads (%)	82.33	82.67	80.92	75.64	76.12	77.47
Clean bases (Gb)	6.27	6.44	7.59	6.35	6.42	6.76
Total unmapped reads (%)	17.67	17.33	19.08	24.36	23.88	22.53
Q20 percentage (%)	97.56	96.72	97.84	97.47	97.35	97.01

### Identification of SSRs and SNPs

**FIGURE 2:**
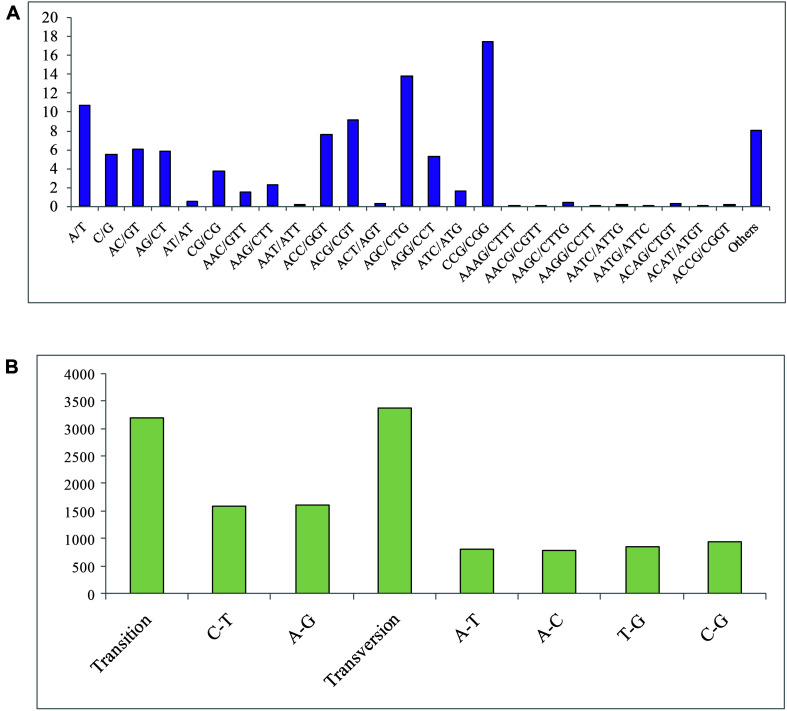
**(A)** Frequency of classified repeat SSR (simple sequence repeats) types. **(B)** SNP (single nucleotide polymorphism) variation types of *P. lilacinum*.

In total, 3,229 SSR markers were detected in 2,997 unigenes, among which, 761 showed SSR > 1. For different motif types of SSRs, trinucleotide (1,895, 58.72%) presented the most frequent nucleotide number, followed by dinucleotide (520, 16.10%), mononucleotide (260, 16.10%), quadnucleotide (35, 1.08%), and other (258, 7.99%) types (Figure 2A). The length of the SSR markers that was primarily distributed in the range of 10 to 30 bp occupied 90.83% of all the SSRs. For motif types of the SSRs, a total of 83 motif types was detected. Moreover, the CCG/CGG type showed the most frequent (563, 17.44%), followed by AGC/CTG (445, 13.78%), and A/T (344, 10.65%) types. All the candidate specific primers designed in this study for SSR markers (primers of 2648 SSRs were successfully obtained) are listed in Supplementary Table 3, and three specific PCR primers were provided for each SSR marker.

In this study, a total of 6,567 SNP sites, including 3,186 transition types and 3,381 transversion types (Figure 2B), was detected. The frequency of SNPs in the *P. lilacinum* transcriptome was 1/3,226 bp, revealing that average 3,226 bp size of unigene could find an SNP site. The A/G and C/T types presented the largest number among all six SNP types, occupying 24.50 and 24.01%, respectively. The other four SNP types (A/T, A/C, G/C, and T/G) exhibited 12.40, 11.91, 14.18, and 13.00%, respectively.

### Analysis of Differentially Expressed Genes

**FIGURE 3:**
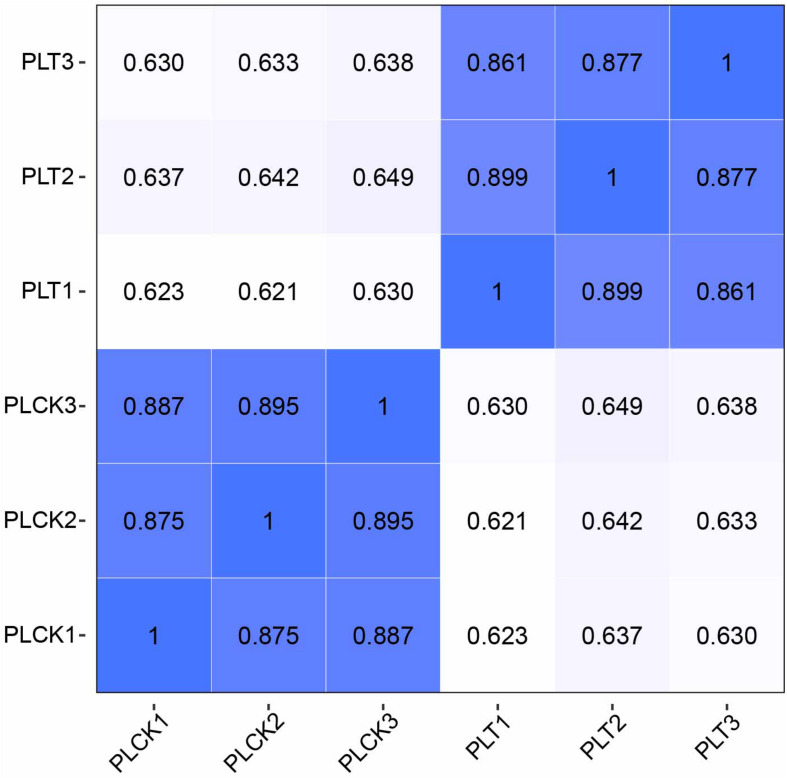
Heatmap of the Pearson’s correlation coefficient for different samples. Greater values and darker-blue squares represent higher similarity between two sequencing libraries. PLT1–PLT3 indicate RNA-seq libraries of *P. lilacinum* infecting *M. incognita* eggs in three biological replicates, respectively; PLCK1–PLCK3 indicate RNA-seq libraries of *P. lilacinum* (controls) in three biological replicates, respectively.

**FIGURE 4:**
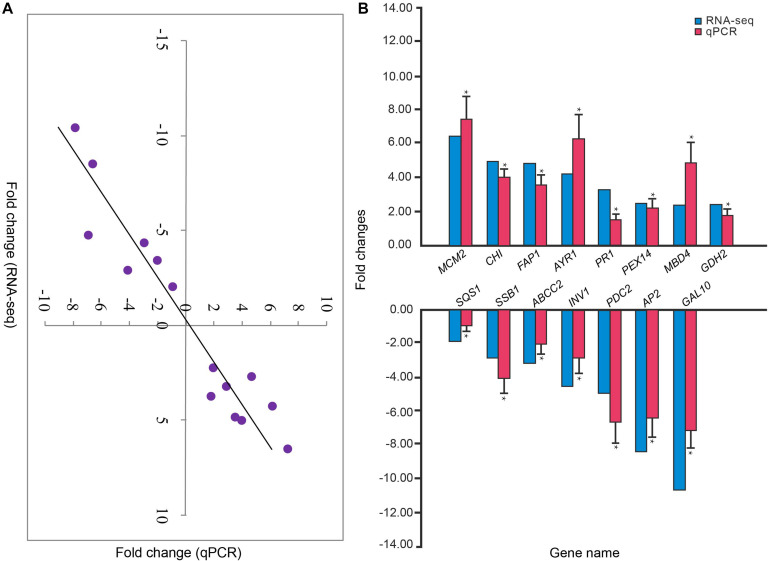
**(A)** Correlation between the relative fold changes in expression from RNA sequencing and qPCR analyses. **(B)** The relative fold changes in expression of 15 genes in *P. lilacinum* infecting *M. incognita* eggs (treatment group) compared to *P. lilacinum* (control group). *Denotes a significant difference in relative fold changes in expression of treatment group compared to control group as determined by unpaired two-tailed *t*-test. *p < 0.01. Three technical replicates were conducted for each of the three biological replicates.

Reproducibility of samples among the three biological replicates was evaluated by calculating Pearson’s correlation coefficient based on the FPKM values of unigenes. Notably, the Pearson’s correlation coefficients exceeded 0.85 between any two replicates from treatment or control data (Figure 3). A high correlation was found among biological replicates, indicating effectiveness of the treatment and absence of accidental errors in individual samples. Moreover, the results of qPCR analysis of the 15 randomly selected DEGs exhibited a close correlation (Pearson correlation coefficient = 0.881, *p* < 0.001) of the fold change in the expression between RNA-seq and qPCR analysis. This indicates reliable RNA-seq and bioinformatic analysis for DEGs (Figures 4A,B).

The current study identified a total of 1,011 DEGs between the control and the treatment groups, including 553 up-regulated DEGs and 458 down-regulated DEGs (Supplementary Table 4). Table 3 summarizes the top 15 up-regulated and 15 down-regulated genes (based on fold change). Among these, three *P. lilacinum* genes that were strongly induced upon infecting *M. incognita* eggs were the genes encoding alpha-galactosidase 1 (*MEL1*), CFEM domain-containing protein (*VFPBJ*), and chitinase (*CHI*). Conversely, three of the most suppressed genes were found to encode extracellular metalloprotease (*EXTM*), alkaline serine protease Alp1 (*ALP1*), and globin-like protein (*GLP*).

**TABLE 3:** Top 15 up- and down-regulated genes in treatment groups compared to the controls, along with corresponding log2Ratios. The significance determination was set as 0.01 *p*-values (Wald test) corrected by the Benjamini–Hochberg method by using the DESeq software.

**Gene ID**	**log2Ratio (Treatment/control)**	**Description**	**Gene name**
Cluster-3954.0	14.23	Alpha-galactosidase 1	*MEL1*
Cluster-4369.0	14.01	CFEM domain-containing protein	*VFPBJ*
Cluster-213.0	12.62	Chitinase	*CHI*
Cluster-700.0	10.98	Hypothetical protein	/
Cluster-3531.290	10.74	Cytochrome P450 oxidoreductase	*CYPs*
Cluster-3958.0	9.99	Integral membrane protein	*ITM*
Cluster-2275.0	9.57	Mitochondrial membrane protein, Mpv17/PMP22	*MPV17*
Cluster-562.0	9.04	Hypothetical protein	/
Cluster-1321.0	8.94	Hypothetical protein	/
Cluster-2838.0	8.73	MFS transporter	*MFS*
Cluster-3531.946	8.27	Putative serine protease EDA2	*EDA2*
Cluster-2609.0	8.20	Alkaline serine protease VCP1	*VCP1*
Cluster-1795.0	8.07	Chitosanase	*CSN*
Cluster-1173.0	8.03	Carbohydrate binding domain-contaning protein	Unavailable
Cluster-1166.0	7.95	Extracellular membrane protein, 8-cysteine region, CFEM	Unavailable
Cluster-2024.0	–12.11	Extracellular metalloprotease	*EXTM*
Cluster-2196.0	–11.49	Alkaline serine protease ALP1	*ALP1*
Cluster-3531.2112	–10.68	Globin-like protein	*GLP*
Cluster-3531.4940	–10.41	Hypothetical protein	/
Cluster-3531.3512	–9.59	Cell surface protein MAS1	*MAS1*
Cluster-1508.0	–9.33	3-carboxymuconate cyclase protein	Unavailable
Cluster-3531.2913	–9.30	Hypothetical protein	/
Cluster-3531.120	–8.97	Serine peptidase	Unavailable
Cluster-3531.5775	–8.88	Proteinase T-like protein	Unavailable
Cluster-2462.0	–8.54	Delta-12 fatty acid desaturase	*FAT*
Cluster-3531.3333	–8.49	Peptidase A4 family protein	Unavailable
Cluster-3531.2292	–8.45	Hypothetical protein	/
Cluster-3035.0	–8.21	Carbohydrate-bindin module family 66 protein	Unavailable
Cluster-808.0	–7.95	Subtilisin-like serine protease precursor	SUB1
Cluster-133.0	–7.92	Alkaline serine protease	*ASPR*

### Functional Enrichment Analysis of Differentially Expressed Genes

A total of 24 GO terms were significantly enriched by DEGs (Table 4). Among these, 15 belonged to biological processes and were primarily involved in ATP production (e.g., GO:0055114: oxidation-reduction process), organic synthesis (e.g., GO:0008610: lipid biosynthetic process), and organic metabolism (e.g., GO:0006629: lipid metabolic process). Moreover, the remaining nine enriched GO terms were involved in molecular functions, and were primarily associated with ATP production and metabolism, catalytic activity, and ribosome function. Examples include oxidoreductase activity (GO:0016491), catalytic activity (GO:0003824), and structural constituent of ribosome (GO:0003735). GO terms involved in oxidoreductase activity were particularly overrepresented.

**TABLE 4:** List of GO terms significantly enriched by differentially expressed genes in the treatment groups compared to the control groups. FDR = FDR-adjusted *p*-values. The Benjamini–Hochberg adjustment was used for FDR correction of the *p*-values (generated by Fisher’s Exact test) of all the GO terms.

**GO id**	**Description**	**Types**	***p*-values**	**FDR**
GO:0055114	Oxidation-reduction process	B	9.57E-14	3.65E-10
GO:0006629	Lipid metabolic process	B	5.24E–09	3.92E–06
GO:0044710	Single-organism metabolic process	B	7.69E–08	5.78E–05
GO:0032787	Monocarboxylic acid metabolic process	B	4.28E–07	9.11E–05
GO:0008610	Lipid biosynthetic process	B	1.17E–06	6.62E–04
GO:0008152	Metabolic process	B	7.16E–06	9.02E–03
GO:0042537	Benzene-containing compound metabolic process	B	3.98E–05	9.71E–03
GO:0043604	Amide biosynthetic process	B	5.72E–05	9.97E–03
GO:0044255	Cellular lipid metabolic process	B	6.11E–05	1.67E–02
GO:0006081	Cellular aldehyde metabolic process	B	2.87E–04	1.31E–02
GO:0044282	Small molecule catabolic process	B	4.34E–04	4.56E–02
GO:0009063	Cellular amino acid catabolic process	B	5.62E–04	4.61E–02
GO:0016054	Organic acid catabolic process	B	6.03E–04	4.66E–02
GO:0044712	Single-organism catabolic process	B	6.17E–04	4.72E–02
GO:0043603	Cellular amide metabolic process	B	7.04E–04	4.88E–02
GO:0016491	Oxidoreductase activity	M	2.78E-11	7.22E–08
GO:0050662	Coenzyme binding	M	8.16E–08	7.67E–05
GO:0016705	Oxidoreductase activity, acting on paired donors, with incorporation or reduction of molecular oxygen	M	3.17E–07	8.95E–05
GO:0003735	Structural constituent of ribosome	M	4.13E–06	1.19E–03
GO:0003824	Catalytic activity	M	5.68E–05	3.44E–03
GO:0048037	Cofactor binding	M	8.15E–05	3.74E–02
GO:0050660	Flavin adenine dinucleotide binding	M	9.91E–05	3.82E–02
GO:0016645	Oxidoreductase activity, acting on the CH-NH group of donors	M	2.68E–04	4.11E–02
GO:0016614	Oxidoreductase activity, acting on CH-OH group of donors	M	3.47E–04	4.57E–02

*B, biological process; M, molecular function.*

In addition, 13 KEGG pathways were found to have significantly enriched DEGs (Table 5), indicating that several metabolic pathways were involved in parasitic behavior of *P. lilacinum* toward *M. incognita* eggs. Clearly, these significantly enriched KEGG pathways are key signaling transducers of aprogressive molecular response to the infection of *M. incognita* eggs. Moreover, these KEGG terms are primarily associated with ribosome function, amino acid degradation and metabolism, ATP production, sugar and organic acid metabolism, as well as DNA replication and repair. These KEGG terms include ribosome (ko03010), valine, leucine, and isoleucine degradation (ko00280); arginine and proline metabolism (ko00330); oxidative phosphorylation (ko00190); galactose metabolism (ko00052); DNA replication (ko03030); and mismatch repair (ko03430), among others.

**TABLE 5:** List of KEGG pathways significantly enriched by differentially expressed genes in the treatment groups compared to the control groups. FDR = FDR-adjusted *p*-values. The Benjamini–Hochberg adjustment was used for FDR correction of the *p*-values (generated by Fisher’s Exact test) of all the KEGG terms.

**KEGG terms**	**ID**	**FDR**
Ribosome	ko03010	1.66E–06
Valine, leucine and isoleucine degradation	ko00280	3.87E–04
Galactose metabolism	ko00052	7.26E–04
Oxidative phosphorylation	ko00190	8.59E–04
DNA replication	ko03030	9.22E–04
Alpha-Linolenic acid metabolism	ko00592	2.56E–03
Arginine and proline metabolism	ko00330	3.92E–03
Other glycan degradation	ko00511	4.25E–03
Phenylalanine metabolism	ko00360	4.89E–03
Fructose and mannose metabolism	ko00051	7.14E–03
Mismatch repair	ko03430	8.67E–03
Glycine, serine and threonine metabolism	ko00260	8.93E–03
Proteasome	ko03050	9.21E–03

## Discussion

In recent years, several studies investigated the mechanism of parasitic behavior of *P. lilacinum* toward the genus *Meloidogyne*. The results showed that rapid growth of *P. lilacinum* mycelium wraps *Meloidogyne* eggs when in contact. The mycelium then infects and kills the eggs (Cadioli et al., 2007). However, little is known about the molecular mechanisms underlying this parasitic behavior of *P. lilacinum* toward *Meloidogyne* eggs, and genetic resources of *P. lilacinum* are also rare. To fill these gaps, this study obtained the transcriptomes of *P. lilacinum* while parasitizing *M. incognita* eggs and compared these results to those of control groups using RNA-seq.

### SNP and SSR Genetic Resources of *P. lilacinum*

Single nucleotide polymorphisms represent single nucleotide mutations occurring at the DNA/RNA level in individuals or samples. In case, these mutations lead to the change in key nucleotides, SNPs may cause the loss of protein function, which can possibly result in the adaptive evolution of fungi (Teixeira et al., 2015; De Fine Licht et al., 2017). Consequently, SNPs are valuable molecular markers for research of molecular mechanisms of host infection, environmental adaptation, and population genetics in *P. lilacinum*. Based on the analysis of transcriptomic data, this study detected molecular markers for *P. lilacinum*. Noteworthy, SSR and SNPs are useful molecular markers for exploring mycological typing techniques and for genetic diversity assessments of fungal biocontrol agents (Schwarzenbach et al., 2007; Piombo et al., 2018). Therefore, this genetic resource is valuable for research on germplasm resource screening of *P. lilacinum* in different regions. Clearly, the availability of SSR and SNPs screened in this study needs further confirmation through PCR and electrophoresis.

### GO Terms Involved in Infection of *M. incognita* Eggs by *P. lilacinum*

This study identified a set of DEGs in *P. lilacinum*, activated by infecting *M. incognita* eggs, indicating that infection of *M. incognita* eggs significantly affects transcriptome-wide gene expression in *P. lilacinum*. Moreover, similar parasitic behavior was also detected in the transcriptomic response of the nematode-trapping fungus *Monacrosporium haptotylum* upon infecting the nematode *Caenorhabditis elegans* (Fekete et al., 2008). These results indicated that the parasitism-dependent morphogenetic transition could be the result of changes in expression and regulation of numerous genes. GO enrichment analysis of DEGs revealed that expression-responsive genes of *P. lilacinum* primarily participated in the oxidation–reduction process, metabolic and biosynthetic processes of organic substance, and catabolic process at molecular level (e.g., small molecules, amino acid, carboxylic acid, and organic acid) upon infecting *M. incognita* eggs. These DEGs were expected to enhance ATP production, carbohydrate metabolism, signal regulation, catabolic process, and biosynthesis of organic substances. Occurrence of these processes contributes to the infection of *M. incognita* eggs by *P. lilacinum*. The genome of the nematode *M. incognita* contains genes related to immune response and antifungal defense (Abad et al., 2008). Overrepresented GO terms are involved in oxidoreductase, ATP production-related process, and energy metabolism. They primarily enhance the detoxification capacity of *P. lilacinum* when faced with the defense response of *M. incognita* eggs (Larriba et al., 2014).

### KEGG Pathways Involved in the Infection of *M. incognita* Eggs by *P. lilacinum*

Several KEGG pathways associated with ATP production and metabolism of nutrients that provide energy (e.g., sugars, fatty acids, and lipids) also get activated, providing further support for the huge requirement for energy by *P. lilacinum* when parasitizing *M. incognita* eggs. Therefore, adequate supply of energy-rich nutrients such as sucrose should enhance the capability of *P. lilacinum* as a biocontrol agent when parasitizing on PPN. Moreover, ribosomes are key organelles involved in protease synthesis. In the current study, the enriched KEGG pathways related to ribosome function indicated that a large number of proteases were synthesized in *P. lilacinum*, which were required for parasitizing on *M. incognita* eggs. Several proteases have been reported to be involved in nematode egg-parasitism, because the eggshell of nematodes is rich in chitin and proteins. Consequently, egg-parasitic fungi need extracellular hydrolases such as protease or chitinase to degrade the eggshell (Yang et al., 2007). Moreover, proteases have been demonstrated to be the key requirement in the processes involved in the parasitism of nematode eggs by fungi, in particular, serine proteases (e.g., subtilisins and serine carboxypeptidases) play a significant role (Lopez-Llorca et al., 2002; Ward et al., 2012). Indeed, a significantly enriched KEGG pathway is associated with the serine metabolism (ko00260 term). This result partially uncovers the molecular mechanisms of serine metabolism during infection of nematode eggs by fungi. More importantly, serine proteases may act as good target for research on functional genomics, and can potentially be applied by testing the invasive ability of fungi through methods of reverse genetics (e.g., RNAi and gene knock-out). Notably, during the infection process, the numbers and expression levels of genes encoding proteases were different among nematode parasitic fungi. For example, compared to other nematode parasitic fungi (e.g., *Pochonia chlamydosporia*), *P. lilacinum* strain 36-1 consisted of more serine proteases and highly expressed genes (e.g., genes encoding serine proteases subfamily S08, S10, S12, S16, and S33) (Xie et al., 2016). This result may partially explain why fungi exhibit varying abilities to penetrate the eggshells of nematodes. Furthermore, amino acids are key components of the egg-shells of PPN. For example, proline residues constitute a large fraction (35%) of amino acids in proteins forming the eggshells of the Tylenchida family (e.g., *Meloidogyne javanica*) (Bird and McClure, 1976). Moreover, nematophagous fungi utilize hydrolytic enzymes to degrade and penetrate nematode egg-shells (Yang et al., 2007; Larriba et al., 2014). In this study, it was found that valine, leucine and isoleucine degradation as well as proteasome pathways (that are involved in proteolytic enzyme activity; Ciechanover, 1994) that participate in infection of *M. incognita* eggs got enriched. This aids in partial identification of the signaling mechanism of eggshell degradation caused by *P. lilacinum* parasitism. As mentioned above, the *M. incognita* genome contains immune- and antifungal-related genes (Larriba et al., 2014), and involvement of this mechanism may damage the DNA of *P. lilacinum*. However, several enriched KEGG pathways involved in DNA replication and repair were detected in this study, thus highlighting the genetic mechanisms underlying self-preservation of *P. lilacinum* as a defense mechanism when infecting *M. incognita* eggs.

### Genes With Strong Expression Response During *P. lilacinum* Infection of *M. incognita* Eggs

This study also focused on individual genes that showed a strong expression response when *P. lilacinum* infected *M. incognita* eggs (i.e., the top 15 genes with fold changes in expression). Noteworthy, the exoglycosidase, namely, alpha-galactosidase catalyzes the hydrolysis of the α-galactoside bond, which is commonly used to eliminate anti-nutritional factors (i.e., the compounds found in most food substances that are poisonous or in other ways limit the availability of nutrients to an organism) in agricultural byproducts and animal feeds (Du et al., 2012). Undeniably, nematode-egg parasitism and plant pathogenicity caused by fungi necessitate the use of a broad set of genes related to detoxification and resistance to oxidative stress, e.g., cytochrome P450 genes (*CYPs*), genes encoding integral membrane proteins, and the CFEM domain-containing protein *VFPBJ* (Chung et al., 1999; Larriba et al., 2014; Zhu et al., 2017). Thus, in *P. lilacinum*, up-regulation of *MEL1* encoding alpha-galactosidase 1 protein, CYPs, and *VFPBJ* likely promotes the resistance to toxicity caused by *M. incognita* eggs. Expression changes in *VCP1* that encode alkaline serine protease and genes encoding carbohydrate binding-related proteins were detected in egg-parasitic nematophagous fungi (i.e., *Pochonia chlamydosporia*) during the colonization of barley roots (Lopez-Llorca et al., 2010). For this, extracellular membrane proteins containing an 8-cysteine domain (CFEM) were identified as signal molecules (either cell-surface receptors or adhesion molecules) in plant pathogenic fungi (Xie et al., 2016). Furthermore, previous studies also found that the genome of *P. lilacinum* strain 36-1 contains significantly more CFEM-containing proteins than other parasitic fungi (e.g., *Metarhizium acridum* and *Magnaporthe oryzae*), and one gene was found to have a predominantly high expression value (Xie et al., 2016). In this study, the expression of one gene that encoded CFEM-containing proteins was found to be strongly up-regulated during the infection of *M. incognita* eggs. This strong effect was found to corroborate the importance of CFEM proteins during the invasion of nematode-eggs. Among the top 15 DEGs detected in this study, several genes were found to encode serine proteases (i.e., *VCP1*, *EDA2*, *SUB1*, *ALP1*, *ASPR*, and genes encoding serine peptidase and proteinase T-like protein) and proteins related to carbohydrate binding (i.e., genes encoding the carbohydrate binding domain-containing protein and carbohydrate-binding module family 66 protein). Noteworthy, changes in the expression of these genes are potentially associated with the parasitic behavior of *P. lilacinum* toward the nematode egg. Chitin is a major component of nematode eggshells (Wharton, 1980) and *P. lilacinum* has been demonstrated to express chitinases during the penetration phase of nematode eggs (Khan et al., 2003, 2004). Furthermore, a previous study also showed co-expression of chitinase-encoding (*CHI*) and genes encoding chitosanases (*CSN*) with *VCP1* by *P. chlamydosporia* in the presence of chitin (Palma-Guerrero et al., 2008). In this study, increased expressions of three of the top 15 DEGs (genes *CHI*, *CSN*, and *VCP1*), possibly indicate their co-involvement in nematode egg parasitism. Furthermore, in the nematophagous fungus *Drechmeria coniospora*, EXTM was identified as an important enzyme involved in the degradation of the nematode body wall (Guo, 2016). According to literature, peptidase A4 family protein also participates in the infection of nematodes by increasing eggshell permeability (Wang et al., 2007; Guo, 2016). Nonetheless, in this study, this gene was down-regulated, which can be explained in terms of inconsistencies between gene expression levels and protein abundance, highlighting the complexity and diversity of molecular mechanisms involved in parasitic behavior of *P. lilacinum*. Delta-12 fatty acid desaturase (FAT) produced by fungi is a key enzyme involved in the synthesis of polyunsaturated fatty acids (Sakuradani et al., 1999). Thus, down-regulation of the *FAT* gene in this study may indicate changes in nutritional requirements of *P. lilacinum* caused by the transition from saprophytism to parasitism. Moreover, despite the fact that a minority of the top 15 DEGs was not linked to *P. lilacinum* parasitism studied herein, this study is the first to identify their key roles in the infection of the *M. incognita* eggs.

## Conclusion

This study identified changes in gene expression of *P. lilacinum* in response to the infection/parasitism of *M. incognita* eggs. A set of SSR and SNPs that are useful for the study of genetic diversity and germplasm screening in *P. lilacinum* was identified. Furthermore, a number of DEGs, functional modules, and pathways involved in *P. lilacinum* parasitism were also identified. These results are helpful toward understanding of the molecular mechanisms involved during the infection of *M. incognita* by *P. lilacinum*. Notably, due to the majority of the unknown for the nematode-parasitic fungus, several obtained results that cannot be reasonably discussed require further explorations. In addition, this study examined the egg infection process of *P. lilacinum* at a single time-point. Because the fungi were at different stages of the infection process at that time point, the transcriptional profiles represent several stages in the infection process.

## Data Availability Statement

The datasets presented in this study can be found in online repositories. The names of the repository/repositories and accession number(s) can be found below: https://www.ncbi.nlm.nih.gov/, SRR10017427-433.

## Author Contributions

L-BL and Q-LZ conceived and designed the study. W-FX, J-LY, X-KM, and Z-GG performed the experiments. Q-LZ, W-FX, J-LY, X-KM, and Z-GG analyzed the data. Q-LZ and W-FX performed statistics. Q-LZ and J-LY drafted the manuscript. L-BL, W-FX, and X-KM revised the manuscript. All authors have read, commented on, and approved the manuscript.

## Conflict of Interest

W-FX, X-KM, and Z-GG was employed by company Kingenta Ecological Engineering Group Co., Ltd. The remaining authors declare that the research was conducted in the absence of any commercial or financial relationships that could be construed as a potential conflict of interest.

## Footnotes

1 http://en.kingenta.com/

2 https://www.ncbi.nlm.nih.gov/genome/281?genome_assembly_id=970664

## Supplementary Material

Supplementary Table 1 Summary for infected rates of *Meloidogyne incognita* eggs with times post exposure to *Purpureocillium lilacinum*.

Supplementary Table 2 Primers used to validate sequencing data by qPCR.

Supplementary Table 3 List of all the specific SSR candidate primers designed by Primer3 software on *Purpureocillium lilacinum* unigenes.

Supplementary Table 4 List of differentially expressed genes (DEGs) between *Meloidogyne*-treated and control groups.
