# Supplementary material for: Understanding the Transcriptional Changes During Infection of Meloidogyne incognita Eggs by the Egg-Parasitic Fungus Purpureocillium lilacinum
Source: Front Microbiol. 2021 Apr 7;12:617710. doi: 10.3389/fmicb.2021.617710 (PMC8058359; doi:10.3389/fmicb.2021.617710)
Supplement: Supplementary Table 1 — Summary for infected rates of Meloidogyne incognita eggs with times post exposure to Purpureocillium lilacinum. [file Table_1.DOCX]

**Supplementary Table 1.** Summary for infected rates of *Meloidogyne incognita* eggs with times post exposure to *Purpureocillium lilacinum*.

| Hours post exposure to *Meloidogyne incognita* eggs | Replicates | Number of eggs | Individuals infected by *Purpureocillium lilacinum* | Infected rates (%) | Means (%) |
| --- | --- | --- | --- | --- | --- |
| 6 pih | R_1 | 100 | 0 | 0 | 0 |
|  | R_2 | 100 | 0 | 0 |  |
|  | R_3 | 100 | 0 | 0 |  |
|  | R_1 | 100 | 10 | 10 |  |
| 18 pih | R_2 | 100 | 9 | 9 | 12±4.4 |
|  | R_3 | 100 | 17 | 17 |  |
| 30 pih | R_1 | 100 | 38 | 38 | 38±2.9 |
|  | R_2 | 100 | 33 | 33 |  |
|  | R_3 | 100 | 33 | 33 |  |
| 42 pih | R_1 | 100 | 56 | 56 | 62±8.1 |
|  | R_2 | 100 | 58 | 58 |  |
|  | R_3 | 100 | 71 | 71 |  |
| 54 pih | R_1 | 100 | 57 | 57 |  |
|  | R_2 | 100 | 74 | 74 | 65±8.6 |
|  | R_3 | 100 | 63 | 63 |  |
|  | R_1 | 100 | 72 | 72 |  |
| 72 pih | R_2 | 100 | 59 | 59 | 66±6.6 |
|  | R_3 | 100 | 67 | 67 |  |

Note：Infection defined by *Purpureocillium lilacinum* mycelium penetrating through the whole individual *Meloidogyne incognita* egg; pih: post-incubation hour
