# Supplementary material for: Understanding the Transcriptional Changes During Infection of Meloidogyne incognita Eggs by the Egg-Parasitic Fungus Purpureocillium lilacinum
Source: Front Microbiol. 2021 Apr 7;12:617710. doi: 10.3389/fmicb.2021.617710 (PMC8058359; doi:10.3389/fmicb.2021.617710)
Supplement: Supplementary Table 2 — Primers used to validate sequencing data by qPCR. [file Table_2.DOCX]

Table S2. Primers used to validate sequencing data by qPCR.

| Gene name | ID | Sequence (5'-3') | Size (bp) |
| --- | --- | --- | --- |
| *MCM2* | Cluster-582.0 | F: CGGTCTTGTCTTCCTCCATT | 86 |
|  |  | R: GAACAGGCAGGCGTGATG |  |
| *CHI* | Cluster-3531.1388 | F: GGCGACGGACTCAATCTC | 149 |
|  |  | R: AGGCTGACAATGGCTCTTAG |  |
| *FAP1* | Cluster-4162.0 | F: GGACGACGATGAAGACAAGAT | 114 |
|  |  | R: AATTCCAGCCGCATACCTT |  |
| *AYR1* | Cluster-1074.0 | F: CTATGTCGCTGCTCACTATGT | 126 |
|  |  | R: CATTGCCACCAAAGGAATAGAC |  |
| *PR1* | Cluster-1707.1 | F: ACCAGAATGATTACCCCTCTCT | 116 |
|  |  | R: TGTCACCACCATCGACTTTG |  |
| *PEX14* | Cluster-4658.0 | F: CTGAACGAGTGCCTGCTT | 109 |
|  |  | R: ACCTGCTGTTGATGTAAGAGAC |  |
| *MBD4* | Cluster-4231.0 | F: CTAACACTGCGAGTCTAC | 113 |
|  |  | R: ATACGGAAAGATTATGAGAGA |  |
| *GDH2* | Cluster-3100.0 | F: TTGACTCGCAATCGTGGTT | 139 |
|  |  | R: GACAGGACTCTTGGAGAATCG |  |
| *SQS1* | Cluster-1134.0 | F: ACCGCATCATTGGCATCA | 106 |
|  |  | R: TCAGCATTATCCTCAAAGTCGTAT |  |
| *SSB1* | Cluster-3556.0 | F: CCAAGTAGCAACGCAGACA | 113 |
|  |  | R: CGGACCAGCAACAGTGAA |  |
| *ABCC2* | Cluster-39.0 | F: CCGATTCAGCCAGGACAT | 126 |
|  |  | R: GCAGCGACACTGTGGATAT |  |
| *INV1* | Cluster-4251.0 | F: CCTCGTCCGATCTCTACCA | 114 |
|  |  | R: AGCCCGAAGTATTGTTCCTATC |  |
| *PDC2* | Cluster-3650.0 | F: ATGCCCAGTAAACCCATTGT | 98 |
|  |  | R: GCTTCTCGCCTCGTTCTC |  |
| *AP2* | Cluster-3531.3333 | F: CACTCTCATTCTTCGGAATCAAG | 92 |
|  |  | R: GGATGGCAGCGTACTTCA |  |
| *GAL10* | Cluster-3531.4940 | F: CAACTGGTGCCTACGACAT | 117 |
|  |  | R: CAATGCCGACCTTCTGGAT |  |
